# Overcoming weeds: breeding herbicide-resistant crops via directed evolution

**DOI:** 10.1093/jxb/erae445

**Published:** 2024-12-03

**Authors:** Liang Ma, Yan Guo

**Affiliations:** State Key Laboratory of Plant Environmental Resilience (SKLPER), College of Biological Sciences, China Agricultural University, Beijing 100193, China; State Key Laboratory of Plant Environmental Resilience (SKLPER), College of Biological Sciences, China Agricultural University, Beijing 100193, China

**Keywords:** Herbicide, HPPD inhibitors, resistance improvement, mesotrione

## Abstract

This article comments on:

**Qian H, Shi H.** 2024. Herbicide-resistant 4-hydroxyphenylpyruvate dioxygenase variants identified via directed evolution. Journal of Experimental Botany **75**, https://doi.org/10.1093/jxb/erae330

This article comments on:

**Qian H, Shi H.** 2024. Herbicide-resistant 4-hydroxyphenylpyruvate dioxygenase variants identified via directed evolution. Journal of Experimental Botany **75**, https://doi.org/10.1093/jxb/erae330

**Increasing resistance of weeds to diverse herbicides has been problematic for agriculture ever since the advent of synthetic herbicides. Creating herbicide-resistant crops via genetic modifications has garnered considerable attention and has currently become a predominant area of research. In a new high-throughput screening study, Qian and Shi (2024) identified four amino acid substitutions in the enzyme 4-hydroxyphenylpyruvate dioxygenase (HPPD) that substantially enhance resistance to the herbicide mesotrione. Their findings demonstrate that some of the combined mutations significantly boost herbicide resistance, while these HPPD mutants retain their native enzyme activities sufficient for normal plant growth and development**.

Genetically modified (GM) crops with tolerance to the broad-spectrum herbicides glyphosate and glufosinate, including maize, soybean, cotton, canola, sugar beet, and alfalfa, have been widely adopted by farmers for efficient weed control, which has significantly impacted agricultural practices globally. However, prolonged and intensive usage of glyphosate has led to increased incidences of herbicide-resistant weeds, which has become a challenge for weed control even with the available herbicide-resistant GM crops (Awika, 2011; Yu *et al*., 2023). Under this scenario, creating crops resistant to alternative herbicides to efficiently manage glyphosate- and glufosinate-resistant weeds is therefore highly desirable (Rüegg *et al*., 2007). One group of widely used effective herbicides is the 4-hydroxyphenylpyruvate dioxygenase (HPPD) inhibitors, and only a few weed species were found to be resistant to this type of herbicides (Ju *et al*., 2023). HPPD inhibitors act by inhibiting the activity of the HPPD enzyme and thus impairing plastoquinone synthesis essential for photosynthesis. Consequently, the *HPPD* gene has been regarded as a potential target for molecular breeding of herbicide-resistant crops for effective weed management in modern agriculture (Ahrens *et al*., 2013; Santucci *et al*., 2017). The research by Qian and Shi (2024) offers a promising approach to creating novel herbicide-resistant crops to address the global challenge of weed control. By employing directed evolution, they identified four single nucleotide mutations in the cotton *HPPD* gene that significantly enhance resistance to mesotrione, a widely used herbicide. Notably, these mutations and their combinations preserved the enzyme’s native function, which ensures elevated herbicide resistance without compromising plant growth and development (Fig. 1).

**Fig. 1.**
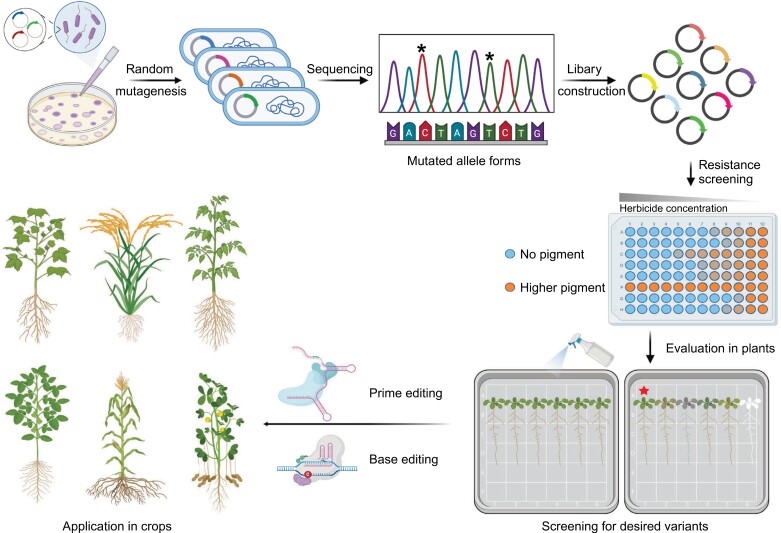
Overview of the directed evolution system. The evolution system combines mutagenesis, high-throughput sequencing, resistance screening, and precise genome editing to develop crops with improved herbicide resistance. Target genes are subjected to random mutagenesis, followed by sequencing to identify mutations that may confer herbicide resistance (mutated alleles marked by asterisks). The library of these mutations is then constructed and subjected to resistance screening using herbicide, where higher pigment levels indicate stronger resistance. Resistant variations are further evaluated in plants and, finally, the desired resistant alleles are introduced into various crop species, such as cotton, rice, tomato, soybean, maize, and peanuts, by base editing and prime editing techniques. The final step involves screening the plants to identify and select the most desirable herbicide-resistant variants for agriculture.

## HPPD inhibitor herbicides

HPPD inhibitors are a wide range of products that are not chemically related but share the same mode of action: inhibiting HPPD activity, which leads indirectly to chlorophyll degradation, leaf bleaching, and ultimately plant death (Ju *et al*., 2023; Ma *et al*., 2024). The first HPPD inhibitor herbicide pyrazolynate was marketed in 1979, and subsequently, numerous commercial products have been launched for weed control in maize, rice, sugarcane, and other cereal crops (Mitchell *et al*., 2001). In recent years, the market share of HPPD inhibitors has been growing, largely due to their effectiveness against weeds that have developed resistance to other herbicides, particularly glyphosate (Ndikuryayo *et al.*, 2017; Jhala *et al.*, 2023).

The herbicidal products targeting HPPD can be classified into three main chemical frameworks: benzoylcyclohexanediones/triketones (mesotrione, sulcotrione, tembotrione, tefuryltrione, and fenquinotrione) (Secor, 1994; Mitchell *et al.*, 2001; Williams and Pataky, 2008); benzoylpyrazoles (pyrazolynate, pyrazoxfen, benzofenap, pyrasulfotole, topramezone, and tolpyralate) (Ikeda and Goh, 1991); and cyclopropylisoxazoles (isoxaflutole) (Pallett *et al*., 1998). This diversity in chemical structures, combined with their shared mechanism of action, makes HPPD inhibitors a critical tool in modern agriculture, especially for managing herbicide-resistant weeds, and mesotrione has emerged as a leading product since its marketing in 2001 (Mitchell *et al*., 2001). The absence of herbicide-resistant crops, however, limits the widespread usage of HPPD-inhibiting herbicides, making HPPD a key target for the molecular design of mesotrione-resistant crops. Recently, Qian and Shi (2024) cloned the *HPPD* gene from cotton with the goal of identifying HPPD variants that provide enhanced resistance to mesotrione while maintaining normal enzyme activity. To achieve this, they first developed a highly efficient screening system using bacteria expressing randomly mutagenized *HPPD* genes that grow on agar plates containing 20 µM mesotrione. The level of mesotrione resistance was assessed by the accumulation of pigment, where higher pigment levels indicated stronger resistance to mesotrione.

## HPPD variations with enhanced herbicide resistance

Multiple previous studies have confirmed that specific mutations in HPPD significantly enhance resistance to HPPD inhibitor herbicides. For instance, the G336W mutation in *Pseudomonas fluorescens* HPPD demonstrated a marked reduction in sensitivity to the inhibitor DKN [2-cyclopropyl-3-(2-mesyl-4-trifluoromethylphenyl)-3-oxo-propanenitrile] and reduced binding affinity for mesotrione, allowing it to retain functionality in the presence of the herbicide (Matringe *et al*., 2005). Similarly, mutations in *Avena sativa* HPPD led to significantly higher resistance to mesotrione (Hawkes *et al*., 2019), and in *Coptis japonica* HPPD resulted in elevated tolerance to destosyl pyrazolate, another potent HPPD inhibitor due to specific mutations (Liang *et al*., 2008). These findings underscore the potential of molecular modifications in HPPD as a powerful approach to developing herbicide-resistant crops while maintaining normal enzyme function.

Directed evolution is a powerful technique for screening multiple HPPD variants, and it has been employed by Qian and Shi (2024) to create enhanced herbicide resistance through random mutagenesis and selection. In this study, four key amino acid substitutions (P329S, T331A, K335E, and G412S) were identified with significantly increased mesotrione resistance. Furthermore, the combination of multiple mutations conferred even higher herbicide resistance than the wild type or individual mutations, indicating that these mutations work synergistically to enhance mesotrione resistance. The authors explain the enhanced mesotrione resistance in terms of decreased binding affinity caused by the mutation-driven structural changes around the helix 11 region in HPPD.

To assess the impact of these mutations on plant growth and development, Qian and Shi (2024) introduced different HPPD variants into an Arabidopsis *hppd* mutant. They found that the combined mutations P329S+K335E (PK), P329S+T331A+K335E (PTK), and P329S+T331A+G412S (PTG) conferred the strongest resistance in Arabidopsis, while retaining normal enzyme activity comparable with the wild-type HPPD. These results highlight the potential of creating specific stacked mutations in crops via gene editing such as base editing and prime editing for enhanced herbicide resistance without compromising crop growth and yield.

## Directed evolution

Directed evolution in *Escherichia coli* is a highly effective approach that mimics the process of natural selection in a controlled laboratory environment to evolve proteins or enzymes with desirable traits. It involves introducing random mutations into the target gene followed by screening or selecting for variants with enhanced or novel functionalities (Packer and Liu, 2015; Wang *et al*., 2022). In their study, Qian and Shi (2024) employed a mutator strain of *E. coli* (XL1-Red), which has impaired DNA repair mechanisms, leading to an elevated mutation rate during DNA replication. This approach generated a diverse library of HPPD variants, each carrying random mutations. Colonies with dark pigmentation were regarded as putative herbicide-resistant mutations since active HPPD forms homogentisic acid from tyrosine, which is converted to a brown pigment. Therefore, colonies with dark pigmentation indicated active and hence mesotrione-insensitive HPPD, as the enzyme was able to function despite the presence of the herbicide. The colonies with clearly enhanced brownish coloration in the presence of mesotrione were isolated, and their plasmids were purified and sequenced to identify the potential herbicide-resistant HPPD mutations.

The application of directed evolution in *E. coli* provides a high-throughput and efficient platform for engineering enzymes with enhanced herbicide resistance. This method has great potential for identifying herbicide-resistant mutations and thus facilitating the development of herbicide-resistant crops, which will greatly contribute to more sustainable agricultural practices.

## Future prospects

Qian and Shi (2024) provide a foundation for the development of an additional herbicide-resistant trait in crops using advanced gene editing tools such as base editing and prime editing. Additionally, the herbicide resistance conferred by these mutations may be further tested across different crops, expanding the utility of HPPD inhibitors for various agricultural applications.

Integrating machine learning techniques into the high-throughput mutant screening system in *E. coli* could enhance the ability to predict and identify new herbicide-resistant variants of HPPD. By investigating and identifying HPPD variants that confer resistance not only to mesotrione but also to other triketone herbicides and related HPPD inhibitors, we can develop more effective variants. Additionally, understanding and identifying potential cross-resistances will be key to broadening agricultural applications, reducing dependence on a single herbicide, and improving overall food security outcomes. This study undoubtedly serves as a preliminary step toward even more promising and exciting research in the future.
